# The Thermodynamic and Gelation Properties of Ovalbumin and Lysozyme

**DOI:** 10.3390/gels11060470

**Published:** 2025-06-19

**Authors:** Lifeng Wang, Rongcheng Li, Siyi Lv, Yulin Liu, Shuaifu Fang, Jingnan Zang, Mingmin Qing, Yujie Chi

**Affiliations:** College of Food Science, Northeast Agricultural University, Harbin 150030, China; lifengwang@neau.edu.cn (L.W.);

**Keywords:** DSC, thermal denaturation, ovalbumin, lysozyme, reversibility

## Abstract

Ovalbumin (OVA) and lysozyme (LYZ) are the predominant globular proteins in egg white and play a crucial role in influencing thermal stability and colloidal behavior. In this study, the thermal and conformational stability of OVA and LYZ under various physicochemical conditions including pH (5–9), protein concentrations (5, 10, and 20%), heating rates (2.5, 5, and 10 °C/min), sugars (sucrose and glucose), and salts (NaCl, KCl, and CaCl_2_) was systematically investigated using differential scanning calorimetry (DSC), aiming to elucidate their behavior within colloidal and gel-forming systems. The denaturation temperatures (T_d_) of OVA and LYZ in water (5% *w*/*v*, 5 °C/min) were 80.22 °C and 77.46 °C, respectively. The T_d_ of LYZ and OVA decreased with protein concentration, heating rate, and CaCl_2_. OVA thermal stability was improved with increasing pH, but the stability of LYZ was decreased. Sugars enhanced the thermal stability of OVA and LYZ. In contrast, NaCl and KCl increased OVA stability but reduced LYZ stability. LYZ exhibited nearly 100% reversibility during the second heating cycle in water. Sugars maintained reversibility at approximately 90% for LYZ. However, the presence of salts diminished the reversibility. In contrast, OVA was completely denatured in water and sugar and salt solutions.

## 1. Introduction

Hen egg white protein (EWP) is a highly excellent source of foods, which is widely used as a key ingredient in numerous food production processes, especially for its functional properties such as emulsification, foaming, and gelation [1,2,3]. In the industrial production of liquid egg whites, the heat pasteurization process is necessary. It serves to sterilize EWP by eliminating pathogenic bacteria and reducing spoilage bacteria (*Salmonella* and *Escherichia coli*) through high-temperature treatment. This is important for ensuring food safety and protecting human health [4,5,6]. However, egg white exhibits limited thermal stability, as it loses fluidity and forms aggregates around 60 °C. And protein aggregation is a common occurrence in egg white production due to its susceptibility to thermal sensitivity [7]. Aggregation is easily induced by external stresses including pH, mechanical vibrations, alkali treatment, pulsed electric fields, and particularly heat treatment [8,9,10,11]. Protein aggregation disrupts the physicochemical properties and functional characteristics of egg whites in food production [12]. During pasteurization egg white protein approaches denaturation, aggregation, and precipitation, which affect the quality of the egg product [7]. Therefore, it is crucial to regulate the thermal stability of egg whites during pasteurization and heat processing [13]. Improving the thermal stability of egg proteins can increase the temperature of heat pasteurization and provide significant benefits for egg manufacturing [14]. Extensive research has focused on the aggregation of egg white proteins, such as the crosslinking of disulfide bonds, and non-covalent hydrophobic and electrostatic interactions [15]. The dominant factors influencing protein aggregation and gel properties are the disulfide bonds and free sulfhydryl groups [12,15,16].

Sample environment including solution pH, ionic strength, and co-solvents, which could promote protein unfolding, enhance hydrophobic interactions, reduce ionic bonds, and disrupt disulfide bonds [17,18]. Egg white is a complex mixture of protein systems with extensive studies focusing on aggregation and gel formation [19]. Comprehensive research has been directed toward understanding the overall properties of the egg white system affected by factors such as salt, sugar, and pH. Consequently, elucidating the thermal properties of key egg white proteins is essential for improving egg product processing and quality control [19,20,21,22].

OVA is the major protein comprising approximately 54% of egg whites. It is a globular phosphoglycoprotein with a molecular mass of 42.8 kDa consisting of 386 amino acid residues containing one disulfide bond and four sulfhydryl groups [23]. OVA is the most important protein in the heat gel formation of egg white aggregation for sulfhydryl groups [24,25]. Lysozyme (LYZ) is approximately 3.4% of egg whites and consists of 129 amino acids with a molecular mass of 14.6 kDa. The tertiary structure is stabilized by four disulfide bonds and no sulfhydryl groups [26]. LYZ has a higher isoelectric point (pI = 10.7) compared to other white proteins, and LYZ is the only alkaline protein in egg white. At natural pH LYZ can bind with other egg white proteins, such as ovomucin, ovalbumin, and ovotransferrin [27]. Meanwhile the antibacterial activity of LYZ has widespread use as a preservative agent in the food industry [28].

OVA and LYZ are the primary factors to affect egg white aggregation and gel formation. Previous studies have demonstrated that OVA and LYZ spontaneously associate with other egg white proteins at extremely low ionic strength even at room temperature [28,29]. In recent years, research has increasingly focused on enhancing the thermal stability and reducing the aggregation of egg white through factors such as pH, salts, and novel technologies [30,31,32,33], and DSC has been used to study the thermal stability of whole egg white system [34,35]. Using specific salts and controlled pH conditions has successfully improved the thermal stability and aggregation behavior of egg white proteins, providing valuable insights for industrial applications. However, there is limited information on the use of differential scanning calorimetry (DSC) to explore the individual protein thermal properties of OVA and LYZ. This study aims to investigate the thermal properties of OVA and LYZ under various conditions, including pH, and the presence of sucrose, glucose, NaCl, KCl, and CaCl_2_ to improve liquid egg white production and explore the potential applications of OVA and LYZ in the food industry.

## 2. Results and Discussion

### 2.1. The Thermal Properties of OVA and LYZ

**Figure 1:**
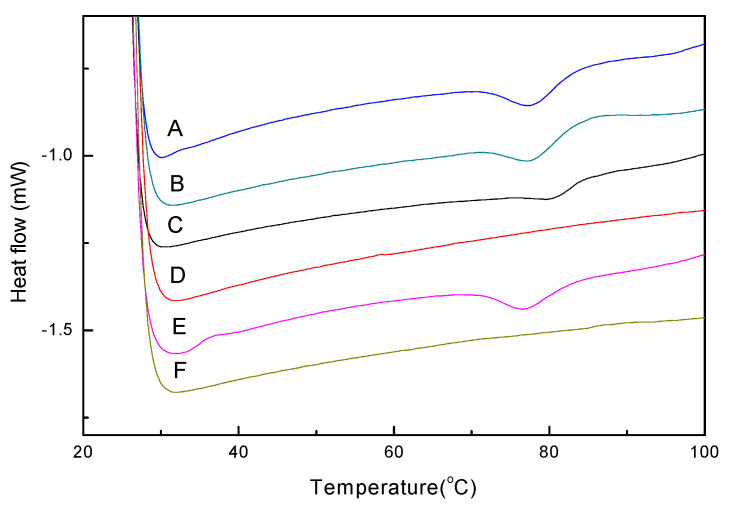
DSC thermograms of OVA, LYZ, and mixture: A. LYZ first cycle (5% *w*/*v*), B. LYZ second cycle (5% *w*/*v*), C. OVA first cycle (5% *w*/*v*), D. OVA second cycle (5% *w*/*v*), E. OVA+LYZ first cycle (10% *w*/*v*), and F. OVA+LYZ second cycle (10% *w*/*v*).

Figure 1 shows the DSC thermograms of OVA, LYZ, and the mixture sample in water. The thermograms of LYZ indicated a single peak and the T_d_ at 77.46 °C. The denaturation peak onset was at 71.28 °C and ended at 83.03 °C with a peak width of 11.75 °C. During the second heating cycle, LYZ exhibited nearly 100% reversibility. The T_d_ remained at 77.46 °C and a consistent enthalpy change (Δ*H* = 7.3 J/g). LYZ exhibited reversibility due to its four disulfide bonds and the absence of free sulfhydryl groups. Upon heating and cooling, the native disulfide linkages are able to reform without generating new bonds or inducing permanent conformational changes, thereby preserving the native tertiary structure [12]. These findings are consistent with previous studies on the thermal behavior of structurally constrained food proteins [36].

The OVA thermograms showed that there was a signal peak at 80.22 °C with denaturation onset at 73.95 °C and endset at 84.23 °C. The peak had a width of 10.28 °C and the enthalpy was 3.5 J/g. Following this, the sample was cooled and reheated for a second heating cycle, but the thermograms indicated no peak, and these results indicate that OVA has no thermal reversibility upon heating. OVA contains four free sulfhydryl groups and one disulfide bond in its structure [37]; when OVA was denatured the structure unfolded and free sulfhydryls formed new disulfide bonds. New disulfide bonds can induce structural changes in OVA, resulting in aggregation and irreversible denaturation [11,38]. This irreversible behavior highlights the propensity of OVA to form thermally induced aggregates and gel networks through disulfide-mediated crosslinking.

Previous research has shown that the structure of egg white proteins can be altered at relatively low temperatures, leading to protein unfolding and the exposure of hydrophobic regions and thiol groups [39]. These structural changes result mainly from the disruption of intramolecular disulfide bonds, exposure of free sulfhydryl groups, and enhancement of hydrophobic interactions, all contributing to irreversible denaturation and aggregation [1]. In the mixed protein system, the thermal stability decreased in the mixed sample. This destabilization may be attributed to the initial unfolding of LYZ, which exposes reactive sites that can interact with unfolded regions of OVA, thereby accelerating OVA’s thermal denaturation [36]. As the protein structure unfolds the exposure of inner hydrophobic and thiol groups increases. These exposed hydrophobic regions and thiol-disulfide interactions drive the unfolding and aggregation of the protein structure [40,41]. Molecular interactions promote protein–protein crosslinking, leading to aggregation and the formation of heat-induced gel-like structures.

### 2.2. Effect of the Heating Rate

The temperature of denaturation (T_d_), onset, endset, and reversibility degree (Rd) of OVA and LYZ at different heating rates (2.5, 5.0, and 10.0 °C/min) are presented in Table 1. The heating rate significantly affected the T_d_ of OVA (*p* < 0.05). The T_d_ of OVA significantly increased with the heating rate (*p* < 0.05), ranging from 78.06 °C to 81.59 °C. However, the enthalpy change (Δ*H* 3.5–3.7 J/g) remained relatively constant across different heating rates. The reversibility of OVA after the second heating cycle was 0%, indicating irreversible denaturation.

The LYZ thermal stability decreased with the heating rate; LYZ exhibited the highest T_d_ (77.79 °C) at 10.0 °C/min and the lowest T_d_ (77.12 °C) at a heating rate of 2.5 °C/min. The Δ*H* of LYZ was slightly altered and ranged from 7.0 to 7.3 J/g. In the second heating cycle the reversibility of LYZ was nearly 100% at all three heating rates. The enthalpy and reversibility of LYZ are less affected by the heating rate. From a kinetic perspective, slower heating rates allow more time for molecular rearrangement, potentially enhancing protein unfolding and dissociation, which may slightly influence the apparent T_d_ values [42,43].

These results indicated that the OVA and LYZ denaturation and thermal aggregation were influenced by heating time. Protein denaturation and aggregation are time-dependent processes that involve protein–protein associations [44]. When protein is heated for extended periods, the protein secondary structure becomes more susceptible to change at lower temperatures [45]. These results eventually lead to a decrease in protein denaturation temperature and aggregation [46]. The heating rate has less effect on the protein denaturation process, so the Δ*H* and protein reversibility were less affected. In contrast, OVA showed a significant increase in T_d_ (*p* < 0.05) due to there being four free sulfhydryl groups in the OVA structure. Free sulfhydryl groups may form new disulfide bonds upon heating, inducing structural changes that result in an elevated T_d_ with an increasing heating rate.

### 2.3. Effect of Protein Concentration on the Thermal Properties

The thermal properties of OVA and LYZ in different protein concentrations are shown in Table 2. The thermal stability of OVA was decreased in higher protein concentrations. The T_d_ of OVA in the protein concentration 200 mg/mL, 10 mg/mL, and 5 mg/mL was 79.93 °C, 80.05 °C, and 80.22 °C. The enthalpy had a less significant effect on protein concentration and the enthalpy at 50 mg/mL and 200 mg/mL was 2.2 J/g and 1.3 J/g, respectively. The T_d_ of LYZ was 74.53 °C, 76.54 °C, and 77.42 °C at protein concentrations of 20%, 10%, and 5%. These values all significantly (*p* < 0.05) increase with decreasing protein concentration. The results indicated that protein concentration could influence the thermal stability of OVA and LYZ [47].

The protein concentration can affect OVA and LYZ heat-induced aggregation. Higher protein concentration samples during heat treatment can aggregate and denature easily [46]. This is because the protein concentration can affect protein reaction with the concentration increasing the reaction of protein, and the results show that protein has less thermal stability with protein concentration increasing [43].

### 2.4. Effect of pH on the Thermal Properties

**Figure 2:**
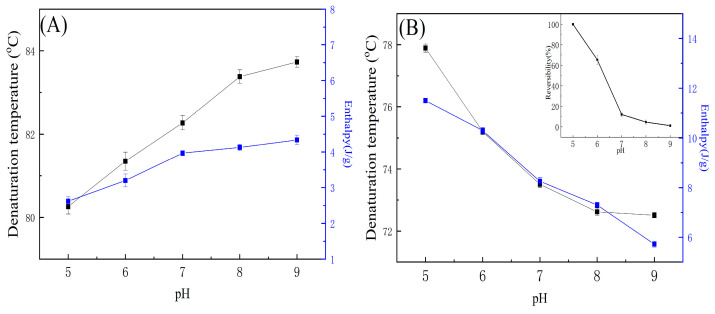
The effect of pH on the thermal properties of OVA and LYZ: (**A**). The effect of pH on OVA. (**B**). The effect of pH on LZY.

As shown in Figure 2, the thermal properties of OVA and LYZ were significantly affected by pH (5.0–9.0). The T_d_ and Δ*H* increased significantly with pH increasing (*p* < 0.05), with T_d_ ranging from 80.26 °C to 83.73 °C and Δ*H* from 2.6 to 4.3 J/g. However, OVA showed no thermal reversibility with pH. This enhanced thermal stability at higher pH may be due to increased electrostatic repulsion and structural rigidity as the pH moves away from OVA’s isoelectric point (pI ≈ 4.5), reducing the tendency for aggregation and unfolding. In contrast, LYZ exhibited decreasing thermal stability with increasing pH. Its T_d_ decreased from 77.89 °C at pH 5.0 to 71.62 °C at pH 9.0, and Δ*H* ranged from 6.6 to 11.1 J/g. Reversibility also dropped dramatically from 100% at pH 5.0 to 1.2% at pH 9.0. This suggests that LYZ undergoes irreversible conformational changes at alkaline pH, likely due to disrupted hydrogen bonding and altered charge distribution, consistent with previous findings [48,49].

pH is an important factor effect protein thermal properties. When the solution pH is close to pI, the net charge of the protein is at a minimum and the protein cooperativity is at a maximum [45]. Therefore, OVA and LYZ have the lowest T_d_ and enthalpy at the pH close to pI. Concurrently, the reversibility rate of LYZ diminishes progressively with the pH. pH affects LYZ net charge, secondary structure, and reversibility rate, and these results are in agreement with previous research that forms irreversible aggregation heated around 80 °C in higher pH [50]. LYZ is a basic protein (pI 10.7), so LYZ tends to associate electrostatically with other acidic proteins [28,51,52].

### 2.5. Effect of Sugars on the Thermal Properties

**Figure 3:**
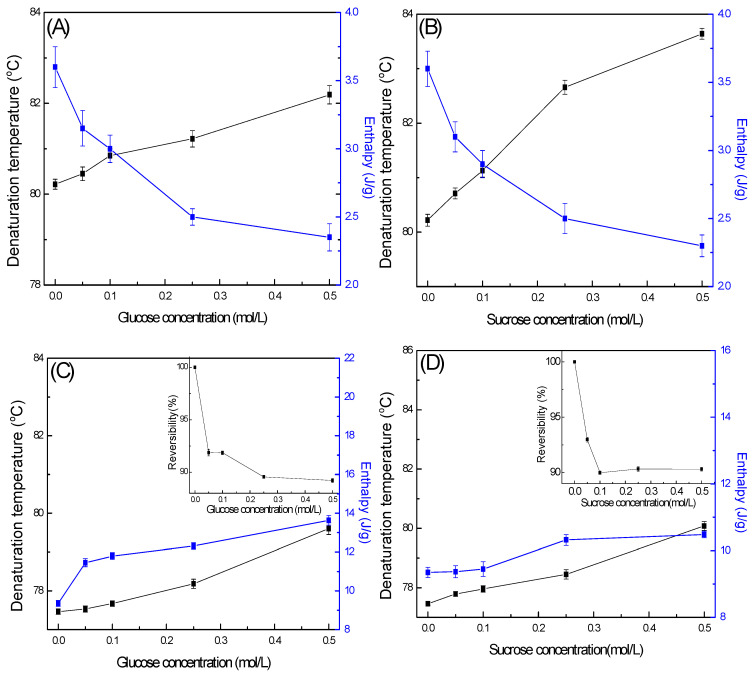
The effect of sugar concentration on the thermal properties of OVA and LYZ: (**A**). The effect of glucose concentration on OVA. (**B**). The effect of sucrose concentration on OVA. (**C**). The effect of glucose concentration on LYZ. (**D**). The effect of sucrose concentration on LYZ.

Figure 3 shows the T_d_ and Δ*H* of OVA and LYZ in glucose and sucrose solutions at concentrations ranging from 0.05 to 0.5 M. Compared to the control (water), LYZ and OVA exhibited significantly higher Td and ΔH values in the presence of sugars. The T_d_ of LYZ increased to 79.61 °C in glucose and 80.08 °C in sucrose. At 0.5 M concentration, Δ*H* increased by 27.45% and 11.39% in the glucose and sucrose solutions, respectively, indicating enhanced thermal stability. Sugars may also form hydrogen bonds with the protein surface, thereby enhancing structural integrity during heating. LYZ exhibited high thermal reversibility in both sugar solutions, with values ranging from 89 to 92% in glucose and 90–93% in sucrose, regardless of sugar concentration.

These results showed that sucrose had a significant increase in the thermal stability of OVA and LYZ than glucose, and the results were concentration-dependent. The values are all significantly (*p* < 0.05) higher than the control sample which was absent of sugar. The stabilizing effect of sucrose and glucose for LYZ was higher than OVA [53,54]. The presence of sugars can influence the entropy release associated with the protein–sugar state, as increased hydrogen bonding contributes to greater structural stability. The observed decrease in Δ*H* is due to the preferential exclusion mechanism of sugar. In this mechanism the protein has less protein conformational stability and decreases the protein flexibility and molecular interactions during protein denaturation, resulting in the protein having higher T_d_ and lower Δ*H* in sugar solution [55,56].

Sugars can improve the thermal stability of LYZ and OVA. At the same concentration, the effect of disaccharides on protein thermal stability was greater than monosaccharides. Sugars can affect the entropy release of the protein–sugar state by enhancing hydrogen bonding, which stabilizes the structure. The observed reduction in Δ*H* may result from the preferential exclusion mechanism, where sugars are excluded from the protein surface. This exclusion limits conformational flexibility and weakens intramolecular interactions during denaturation, leading to a higher T_d_ and lower Δ*H* in sugar solutions. Sugar molecules were preferentially or weakly excluded from the protein surface and preserved the native protein hydration shell [57]. The cohesive force increased as sugar concentration increased, which increased the energy required for cavity formation for the associated structures in the solvent [58,59].

### 2.6. Effect of Salts on the Thermal Properties

#### 2.6.1. Effect of Salts on Thermal Properties of OVA

**Figure 4:**
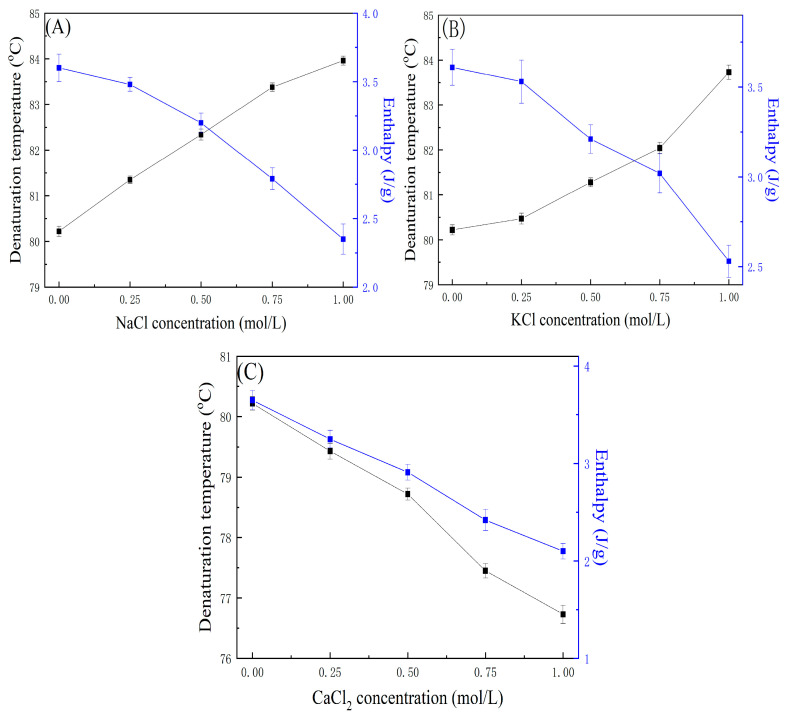
The effect of (**A**) NaCl, (**B**) KCl, and (**C**) CaCl_2_ concentration on the thermal properties of OVA.

Ions can influence the net charge, hydration shell, and electrostatic interactions of proteins, thereby affecting their structure and thermal stability [60]. As shown in Figure 4, the thermal behavior of OVA was evaluated in the presence of different concentrations (0.25–1.0 M) of NaCl, KCl, and CaCl_2_. In comparison to the sample absent of salt, the thermal stability of OVA had a significant improvement in NaCl and KCl solution (1.0 M) with the T_d_ being 84.09 °C and 83.96 °C. However, in the CaCl_2_ solution, the stability of OVA had a significant decrease and T_d_ was reduced to 76.70 °C. When the samples were reheated, there was no reversibility in the second cycle. In comparison to the absent CaCl_2_, the Δ*H* of OVA decreased by 42% in 1.0 M CaCl_2_.

NaCl and KCl have a stabilizing effect on the thermal stability of OVA which was concentration-depended. The enthalpy of OVA was decreased by about 29.5% and 24.3% in 1.0 M NaCl and KCl solution. NaCl and KCl can change electrostatic interactions between protein molecules by altering surface charge. Additionally, salts may enhance hydrophobic interactions among proteins, thereby influencing their structural stability [61].

The thermal stability of OVA increased with rising concentrations of NaCl and KCl, as Na^+^ and K^+^ ions can alter the net surface charge of the protein and change intermolecular interactions. Less stable regions within the protein fold may act as nucleation sites for further unfolding under thermal stress [62]. In contrast, CaCl_2_ can form calcium bridges with the protein, leading to structural alterations. As a result, CaCl_2_ exerts a stronger destabilizing effect on protein conformation and thermal stability [63]. Cations with high charge density tightly bind water molecules in a structured hydration shell that limits their ability to form strong hydrogen bonds with the surrounding water. Under these conditions, anion-specific effects become more pronounced, as anions compete for the limited hydrogen-bonding sites available on the remaining unbound water molecules at the hydration shell interface [64].

When OVA was reheated in the NaCl and KCl solutions, the reversibility of OVA was 0%. The salt solution could increase the thermal stability and affect the denaturation mechanism. During the protein denaturing process the free thiol interchanges with disulfide, forming new disulfide bonds [65]. Salt can stabilize egg white thermal properties and combining it with sugar can have a synergistic effect on keeping the denatured protein in solution [14,54].

#### 2.6.2. Effect of Salts on Thermal Properties of LYZ

**Figure 5:**
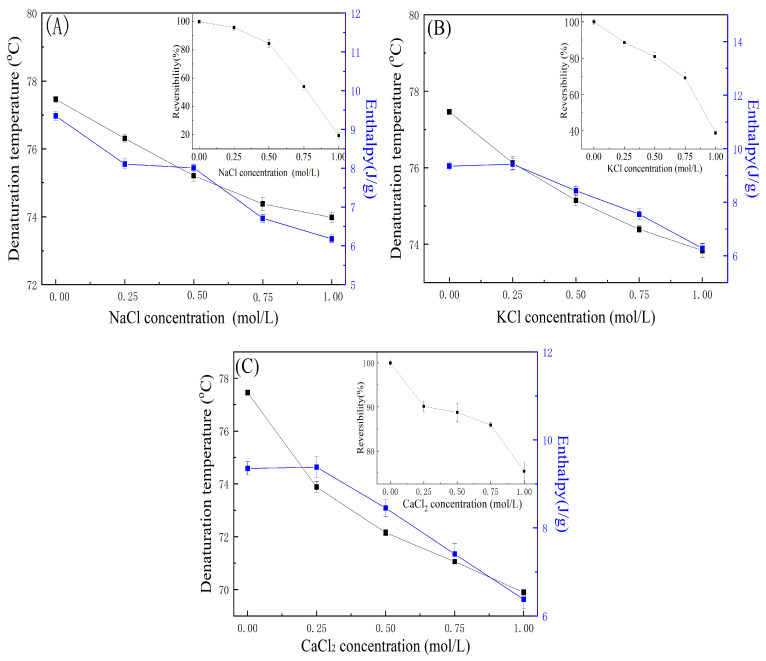
The effect of (**A**) NaCl, (**B**) KCl, and (**C**) CaCl_2_ concentration on the thermal properties of LYZ.

Figure 5 shows the DSC results of LYZ heated in NaCl, KCl, and CaCl_2_ solutions at different salt concentrations. The T_d_ of LYZ significantly decreased as the salts concentration. In comparison with the salt-free sample (77.46 °C), the T_d_ of LYZ decreased to 73.92 °C (1.0 M NaCl), 74.23 °C (1.0 M KCl), and 68.89 °C (1.0 M CaCl_2_) when the salt solution concentration reached 1.0 M. In salt solution, the enthalpy of LYZ decreases with salt concentration, the decreasing trend and value are relatively consistent. The enthalpy of LYZ ranged from 6.2 to 6.4 J/g in 1.0 M solution of three salts. There was a significant effect on the thermodynamic negative stability and the enthalpy decreased with salt concentration.

These results indicate that KCl, NaCl, and CaCl_2_ have a significant impact on the thermal stability of LYZ in salt solutions, and the thermal destabilization is observed with the order Ca^2+^ > K^+^ > Na^+^. The result is in agreement with previous research [66]. And the order of the effect on LYZ is opposite to the traditional Hofmeister series [67,68].

Salts can affect the thermal stability, T_d_, Δ*H*, and reversibility degree of OVA and LYZ, as cations can alter protein electrostatic interactions by changing the net charge on the protein surface. In high-concentration salt solutions, the net surface charge is reduced, resulting in weakened electrostatic interactions between macromolecules and the suppression of coacervation. In high salt solutions, cations can influence surface charge density serving as nucleation centers for further protein unfolding due to their inherent instability within the folded structure [69]. In the second heating cycle, the reversibility of LYZ was decreased with salt concentration, and the reversibility of LYZ was decreased with salt concentration. The reversibility of LYZ in three salt solutions (0.25 M) was about 90%. When the salts concentration increased to 1.0 M, the reversibility was decreased to 19.41% in NaCl (1.0 M), 35.85% in KCl (1.0 M), and 75.39% in CaCl_2_ (1.0 M).

All salts exhibited a similar trend in reducing the reversibility of LYZ; however, CaCl_2_ resulted in a higher degree of LYZ reversibility compared to NaCl and KCl. The observed trend followed the order Ca^2^^+^ > K^+^ > Na^+^, consistent with the Hofmeister series. Salts have a similar effect on reducing LYZ reversibility, but CaCl_2_ showed higher values of LYZ reversibility than NaCl and KCl. And the following trend appeared as Ca^2+^ > K^+^ > Na^+^ which follows the Hofmeister series, and cation can affect the charge on the protein surface charge and affect the protein–protein encounters and denature to decrease the reversibility of LYZ. In the solution, the presence of cation can increase the energy barrier for association and effectively screen charge interactions in the unfolded state, and this results in a reduction in the thermal reversibility of OVA and LYZ [70].

### 2.7. Structural Characteristics of OVA and LYZ

**Figure 6:**
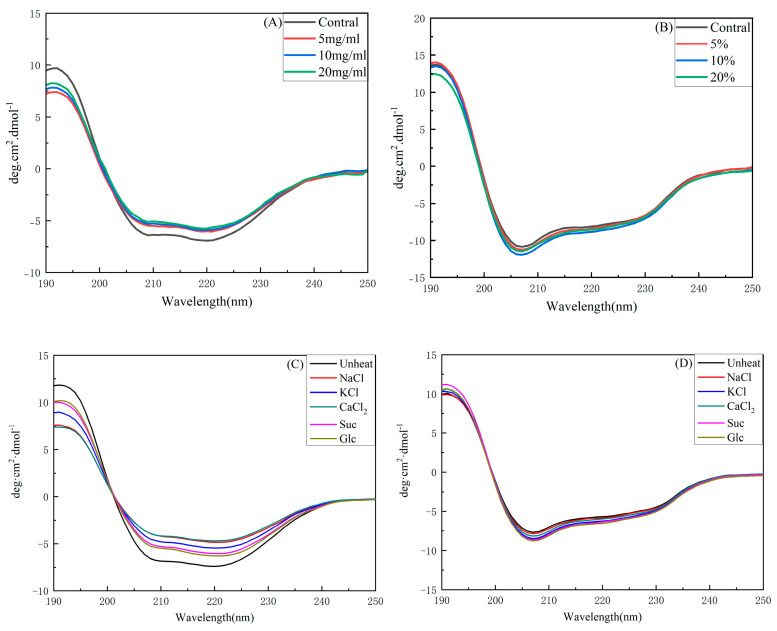
The influence of the salts, sugars, and protein concentration on the secondary structure from CD analysis: (**A**) The effect of concentration on OVA, (**B**) the effect of concentration on OVA, (**C**) the effect of salt and sugars on OVA, and (**D**) the effect of salt and sugars on LYZ.

The secondary structural changes in OVA and LYZ are shown in Figure 6. OVA exhibited two negative peaks at approximately 208 and 222 nm and a positive peak at 190 nm, indicating the presence of α-helical structures. Additionally, a positive band around 195 nm and a negative band at 218 nm were also observed, which are indicative of β-sheet structures [71]. When OVA was denatured the value of peaks was decreased, which means that the OVA had lost the protein secondary structure. Figure 6A,B indicate that OVA at different concentrations affects protein structural changes upon heating, as evidenced by the decreased absorption peaks at 190, 208, and 222 nm in the circular dichroism (CD) spectra. The results showed protein denaturation and conformational change after heating. With increasing protein concentration the α-helical content decreased and the random structure showed an increasing trend. In contrast, the secondary structure of LYZ remained relatively stable, with protein concentration having minimal impact on the protein secondary structure after heating [72,73].

The CD spectra of salt solutions (NaCl, KCl, and CaCl_2_) and water were similar; however, in the sugar solution (sucrose and glucose), the curve was more similar to that of the unheated sample, suggesting that sugar can protect the OVA structure better. In sugar solution the protein exhibited more α-helical and less random structure. The LYZ CD spectra showed two negative peaks at approximately 208 and 220 nm, and on the side had positive peaks at around 190 and 240 nm [74]. The similarity of these curves suggests that LYZ possesses excellent thermal stability and reversibility. In sugar solution the peak value was increased at the α-helical and β-sheet structures indicating that LYZ has enhanced thermal stability in the sugar solution. In contrast salts (NaCl, KCl, and CaCl_2_) decreased the content of α-helical and increased the random structure and this result shows that the salts cannot stabilize the secondary structure [75]. This result is consistent with the DSC results, which showed that sugars help stabilize α-helical and β-sheet structures, thereby enhancing protein thermal stability.

## 3. Conclusions

This study systematically elucidates the effects of pH, salts, sugars, heating rate, and protein concentration on the thermal properties of OVA and LYZ. The results demonstrate that pH modulates the thermal stability of OVA and LYZ differently. Increased protein concentration and slower heating rates were associated with reduced thermal stability, suggesting enhanced molecular interactions and aggregation under these conditions. Sugars (monosaccharides and disaccharides) significantly improved the thermal stability of proteins in a concentration-dependent factor, providing insights into their protective roles in heat processing. Regarding ionic effects, Na^+^ and K^+^ ions markedly increased OVA thermal stability, whereas Ca^2^^+^ decreased it due to calcium-induced structural alterations and possible bridging effects. LYZ exhibited reduced T_d_ in all the salt solutions, with a clear concentration-dependent decline in reversibility ranging from 20% to 90%. OVA displayed complete and irreversible denaturation across all the tested conditions, regardless of pH, salts, and sugars. These results show extensive secondary structural loss, sulfhydryl-disulfide exchange, and hydrophobic interactions, supporting the notion that OVA has a strong propensity for heat-induced aggregation and network formation, positioning it as a promising gel-forming protein. LYZ retained a higher degree of structural integrity and reversibility following thermal treatment, indicating limited capacity to participate in heat-induced gelation. These results of OVA and LYZ offer valuable theoretical support for the rational design and optimization of protein-based gel systems in food and colloidal applications.

## 4. Materials and Methods

### 4.1. Materials

Ovalbumin (product A5503 approximately 98% purity as per manufacturer) and lysozyme (product L6876 approximately 90% purity as per manufacturer) of hen white protein obtained from Sigma-Aldrich Co. (St. Louis, MO, USA) were used. Sugars, NaCl, KCl, and CaCl_2_ were from Titan Technology Co., Ltd. (Tianjin, China). All the reagents were of analytical grade.

### 4.2. Sample Preparation

Solutions containing OVA and LYZ were prepared by dissolving the proteins in H_2_O to achieve a concentration of 50 mg/mL. Preliminary experiments demonstrated that a concentration of 50 mg/mL for each protein was necessary to achieve satisfactory DSC sensitivity. The samples underwent gentle agitation and aliquots (10 μL) of the solutions were subsequently transferred into DSC pans for analysis.

#### 4.2.1. Effect of pH

The OVA and LYZ were prepared in 0.1 mol/L phosphate buffers ranging from pH 5 to pH 9 and the protein concentration was 50 mg/mL, pH was adjustment with NaOH (1 mol/L) and HCl (1 mol/L) was determined according to the method of Alli [36]. The phosphate buffers were prepared with Na_2_HPO_4_ and NaH_2_PO_4_ as reagents.

#### 4.2.2. Effect of Salts

To investigate the effect of Na^+^, K^+^, and Ca^2+^ on the thermal properties of OVA and LYZ, OVA and LYZ were dissolved in solutions of NaCl, KCl, and CaCl_2_ with pH ranging from 7.0 to 7.1 and different concentrations (0.25, 0.50, 0.75, and 1.0 M), the final protein concentration was 50 mg/mL.

#### 4.2.3. Effect of Sugars

To investigate the effect of sugars on the thermal properties of OVA and LYZ, OVA and LYZ were dissolved in sucrose and glucose solutions with concentrations 0.05, 0.1, 0.25, and 0.5 M, the final protein concentration was 50 mg/mL.

#### 4.2.4. Effect of Heating Rate

To investigate the effect of heating rate on the thermal properties OVA and LYZ were dissolved separately in water (pH 7.0) at a concentration of 50 mg/mL. The samples were heated at different heating rates (2.5, 5.0, and 10.0 °C/min), respectively.

#### 4.2.5. Effect of Protein Concentration

The method to investigate the effect of different protein concentrations was determined according to the method of Alli [36]: OVA and LYZ were dissolved in water at concentrations of 50 mg/mL, 100 mg/mL, and 200 mg/mL, respectively. And then samples were heated at a heating rate of 5.0 °C/min.

#### 4.2.6. Differential Scanning Calorimetry (DSC)

All samples were heat-treated and analyzed by DSC (STARe SYSTEM, METTLER TOLEDO, Greifensee, Switzerland). Each sample was heated from 25 to 100 °C at a heating rate of 5 °C/min. The peak temperature of protein denaturation (T_d_) and heat of transition of enthalpy (Δ*H*) (area underneath peak) were calculated from each thermal graph. After heating, the samples were cooled to 25 °C in DSC and reheated the second cycle under the same conditions. The reversibility rate of OVA and LYZ was determined from the ratio of the first and second endothermal peaks [76]. All DSC sample experiments were performed in triplicate.

#### 4.2.7. Circular Dichroism Spectrum

Circular dichroism (CD) spectra were taken using a chirascan (V100) spectropolarimeter (Applied Photophysics Ltd., Surrey, UK). The circular dichroism spectra of the OVA and LYZ samples (50 mg/mL) in water and different salt (NaCl, KCl, and CaCl_2_) and sugar (glucose and sucrose) solutions were collected at the 0.1 mol/L concentration. The samples were recorded at 25 °C in the range from 190 to 250 nm with a spectral resolution of 0.1 nm, and the protein concentration was 0.2 mg/mL. The scan speed was 100 nm/s and the response time was 0.125 s with the bandwidth of 1 nm. Quartz cells with an optical path of 0.1 cm were used. Typically, 16 scans were accumulated and subsequently averaged. A total of three spectra were accumulated and averaged.

#### 4.2.8. Statistical Analysis

All the experiments were performed in triplicate. The data were analyzed using a One-Way Analysis of Variance (ANOVA) test, and the means were compared using Fisher’s least significant difference test (*p* < 0.05).

## Figures and Tables

**Table 1:** The effect of heating rate on the thermal properties of OVA and LYZ.

Rate (°C/min)		OVA					LYZ			
	Onset (°C)	Td (°C)	Endset (°C)	Δ*H* (J/g)	Rd (%)	Onset (°C)	Td (°C)	Endset (°C)	Δ*H* (J/g)	Rd (%)
2.5	75.06 ± 0.15 ^a^	78.06 ± 0.19 ^a^	81.39 ± 0.21 ^a^	3.5 ± 0.2 ^a^	0	66.65 ± 0.13 ^a^	76.18 ± 0.12 ^a^	79.60 ± 0.16 ^a^	7.0 ± 0.1 ^a^	100
5	75.68 ± 0.17 ^b^	80.22 ± 0.11 ^b^	84.38 ± 0.15 ^b^	3.5 ± 0.1 ^b^	0	71.50 ± 0.18 ^b^	77.46 ± 0.05 ^b^	82.45 ± 0.04 ^b^	7.3 ± 0.1 ^b^	100
10	77.13 ± 0.18 ^c^	81.59 ± 0.19 ^c^	85.51 ± 0.17 ^c^	3.7 ± 0.1 ^b^	0	71.55 ± 0.11 ^b^	77.68 ± 0.12 ^c^	83.40 ± 0.13 ^c^	7.4 ± 0.1 ^b^	100

Different small letters in the same column indicate that values are significantly different (*p* < 0.05).

**Table 2:** The effect of protein concentration on the thermal properties of OVA and LYZ.

Protein mg/mL		OVA					LYZ			
	Onset (°C)	Td (°C)	Endset (°C)	Δ*H* (J/g)	Rd (%)	Onset (°C)	Td (°C)	Endset (°C)	Δ*H* (J/g)	Rd (%)
50	75.68 ± 0.17 ^a^	80.22 ± 0.11 ^a^	84.38 ± 0.15 ^a^	3.5 ± 0.1 ^a^	0	71.50 ± 0.18 ^a^	77.46 ± 0.05 ^a^	82.45 ± 0.04 ^a^	7.3 ± 0.1 ^a^	100
100	74.06 ± 0.12 ^b^	80.05 ± 0.03 ^b^	83.73 ± 0.13 ^b^	3.2 ± 0.1 ^b^	0	70.55 ± 0.22 ^b^	76.51 ± 0.07 ^b^	81.97 ± 0.11 ^b^	7.2 ± 0.1 ^a^	100
200	73.63 ± 0.11 ^c^	79.93 ± 0.06 ^b^	83.57 ± 0.03 ^c^	3.0 ± 0.1 ^c^	0	68.93 ± 0.16 ^c^	74.53 ± 0.03 ^c^	79.90 ± 0.13 ^c^	7.1 ± 0.1 ^a^	100

Different small letters in the same column indicate that values are significantly different (*p* < 0.05).

## Data Availability

The original contributions presented in this study are included in the article. Further inquiries can be directed to the corresponding author.

## References

[1] Mine Y., Ustunol Z. (2014). Egg proteins. Applied Food Protein Chemistry.

[2] Abeyrathne E.D.N.S., Lee H.Y., Ahn D.U. (2013). Egg white proteins and their potential use in food processing or as nutraceutical and pharmaceutical agents—A review. Poult.

[3] Wu C., Wang T., Ren C., Ma W., Wu D., Xu X., Wang L.S., Du M. (2021). Advancement of food-derived mixed protein systems: Interactions, aggregations, and functional properties. Compr. Rev. Food Sci. Food Saf..

[4] Yang Y., Geveke D.J. (2020). Shell egg pasteurization using radio frequency in combination with hot air or hot water. Food Microbiol..

[5] Keener K.M. (2017). Shell Egg Pasteurization. Egg Innovations and Strategies for Improvements.

[6] Georgescu N., Apostol L., Gherendi F. (2017). Inactivation of Salmonella enterica serovar Typhimurium on egg surface, by direct and indirect treatments with cold atmospheric plasma. Food Control.

[7] Mizutani K., Chen Y., Yamashita H., Hirose M., Aibara S. (2006). Thermostabilization of Ovotransferrin by Anions for Pasteurization of Liquid Egg White. Biosci. Biotechnol. Biochem..

[8] Liu J., Zhu K., Ye T., Wan S., Wang Y., Wang D., Li B., Wang C. (2013). Influence of konjac glucomannan on gelling properties and water state in egg white protein gel. Food Res. Int..

[9] Ferreira M., Hofer C., Raemy A. (1997). A calorimetric study of egg white proteins. J. Therm. Anal..

[10] Plancken I.V.D., Loey A.V., Hendrickx M.E. (2007). Foaming properties of egg white proteins affected by heat or high pressure treatment. J. Food Eng..

[11] Gharbi N., Labbafi M. (2018). Effect of processing on aggregation mechanism of egg white proteins. Food Chem..

[12] Iesel V.D.P., Van Loey A., Hendrickx M.E.G. (2005). Changes in Sulfhydryl Content of Egg White Proteins Due to Heat and Pressure Treatment. J. Agric. Food Chem..

[13] Hong T., Iwashita K., Handa A., Shiraki K. (2017). Arginine prevents thermal aggregation of hen egg white proteins. Food Res. Int..

[14] Campbell L., Raikos V., Euston S.R. (2003). Modification of functional properties of egg-white proteins. Nahr. Food.

[15] Iwashita K., Handa A., Shiraki K. (2018). Co-aggregation of ovotransferrin and lysozyme. Food Hydrocoll..

[16] Hong T.K.J.S.A.K. (2021). Aggregation of hen egg white proteins with additives during agitation. LWT Food Sci. Technol..

[17] Handa A., Takahashi K., Kuroda N., Froning G.W. (2010). Heat-induced Egg White Gels as Affected by pH. J. Food Sci..

[18] Mine Y., Noutomi T., Haga N. (1991). Thermally induced changes in egg white proteins. Ital. J. Zool..

[19] Matsuda T., Watanabe K., Sato Y. (2010). Heat-Induced Aggregation of Egg White Proteins as Studied by Vertical Flat-Sheet Polyacrylamide Gel Electrophoresis. J. Food Ence.

[20] Iwashita K., Handa A., Shiraki K. (2017). Co-aggregation of ovalbumin and lysozyme. Food Hydrocoll..

[21] Totosaus A., José G.M., Salazar J.A., Guerrero I. (2010). A review of physical and chemical protein-gel induction. Int. J. Food Sci. Technol..

[22] Zhang Z., Yang Y., Tang X., Chen Y., You Y. (2016). Chemical forces study of heat-induced myofibrillar protein gel as affected by partial substitution of NaCl with KCl, MgCl2 and CaCl2. CyTA J. Food.

[23] Huntington J.A., Stein P.E. (2001). Structure and properties of ovalbumin. J. Chromatogr. B Biomed. Sci. Appl..

[24] Kawachi Y., Kameyama R., Handa A., Takahashi N., Tanaka N. (2013). Role of the N-Terminal Amphiphilic Region of Ovalbumin during Heat-Induced Aggregation and Gelation. J. Agric. Food Chem..

[25] Yamashita H., Ishibashi J., Hong Y.H., Hirose M. (1998). Involvement of Ovotransferrin in the Thermally Induced Gelation of Egg White at around 65 °C. Biosci. Biotechnol. Biochem..

[26] Clark E.D.B., Hevehan D., Szela S., Maachupalli-Reddy J. (2010). Oxidative renaturation of hen egg-white lysozyme. Folding vs aggregation. Biotechnol. Prog..

[27] Li-Chan E.C., Kim H.O., Mine Y. (2008). Structure and chemical composition of eggs. Egg Bioscience and Biotechnology.

[28] Le Floch-Fouéré C., Pezennec S., Lechevalier V., Beaufils S., Desbat B., Pézolet M., Renault A. (2009). Synergy between ovalbumin and lysozyme leads to non-additive interfacial and foaming properties of mixtures. Food Hydrocoll..

[29] Yu S., Yao P., Jiang M., Zhang G. (2010). Nanogels prepared by self-assembly of oppositely charged globular proteins. Biopolymers.

[30] Alavi F., Emam-Djomeh Z., Chen L. (2020). Acid-induced gelation of thermal co-aggregates from egg white and hempseed protein: Impact of microbial transglutaminase on mechanical and microstructural properties of gels. Food Hydrocoll..

[31] Mohammadi Nafchi A., Tabatabaei R.H., Pashania B., Rajabi H.Z., Karim A.A. (2013). Effects of ascorbic acid and sugars on solubility, thermal, and mechanical properties of egg white protein gels. Int. J. Biol. Macromol..

[32] Zhang-Yi C., Yan Z., Yong-Gang T.U., Jian-Ke L.I., Xu-Ying L., Jun-Jie W., Wen-Hui D. (2014). Research progress in the gelation mechanism of egg white proteins. Sci. Technol. Food Ind..

[33] Jin H., Chen J., Zhang J., Sheng L. (2020). Impact of phosphates on heat-induced egg white gel properties: Texture, water state, micro-rheology and microstructure. Food Hydrocoll..

[34] Llave Y., Fukuda S., Fukuoka M., Shibata-Ishiwatari N., Sakai N. (2018). Analysis of color changes in chicken egg yolks and whites based on degree of thermal protein denaturation during ohmic heating and water bath treatment. J. Food Eng..

[35] Shibata-Ishiwatari N., Takagi T., Fukuoka M., Sakai N. (2018). Kinetic Studies on the Effect of Salt on the Thermal Denaturation of Egg Constituents. Jpn. J. Food Eng..

[36] Boye J.I., Alli I. (2000). Thermal denaturation of mixtures of α-lactalbumin and β-lactoglobulin: A differential scanning calorimetric study. Food Res. Int..

[37] Liu T., Zhao Y., Wu N., Chen S., Xu M., Du H., Yao Y., Tu Y. (2022). Egg white protein-based delivery system for bioactive substances: A review. Crit. Rev. Food Sci. Nutr..

[38] Ding L., Xia M., Zeng Q., Zhao Q., Cai Z., Zhu Z. (2022). Foaming properties and aggregation mechanism of egg white protein with different physical treatments. LWT.

[39] Kuang J., Hamon P., Lechevalier V., Saurel R. (2023). Thermal Behavior of Pea and Egg White Protein Mixtures. Foods.

[40] Mine Y. (1995). Recent advances in the understanding of egg white protein functionality. Trends Food Sci. Technol..

[41] Hirose M. (1993). Molten globule state of food proteins. Trends Food Sci. Technol..

[42] Hoffmann M.A.M., Miltenburg J.C.V., Van Mil P.J.J.M. (1997). The suitability of scanning calorimetry to investigate slow irreversible protein denaturation. Thermochim. Acta.

[43] Relkin P. (1994). Differential scanning calorimetry: A useful tool for studying protein denaturation. Thermochim. Acta.

[44] Mession J.L., Sok N., Assifaoui A., Saurel R. (2013). Thermal Denaturation of Pea Globulins (*Pisum sativum* L.)—Molecular Interactions Leading to Heat-Induced Protein Aggregation. J. Agric. Food Chem..

[45] Relkin P. (1996). Thermal unfolding of beta-lactoglobulin, alpha-lactalbumin, and bovine serum albumin. A thermodynamic approach. Crit. Rev. Food Sci. Nutr..

[46] Haug I.J., Skar H.M., Vegarud G.E., Langsrud T., Draget K.I. (2009). Electrostatic effects on β-lactoglobulin transitions during heat denaturation as studied by differential scanning calorimetry. Food Hydrocoll..

[47] Cao X., Li J., Yang X., Duan Y., Liu Y., Wang C. (2008). Nonisothermal kinetic analysis of the effect of protein concentration on BSA aggregation at high concentration by DSC. Thermochim. Acta.

[48] Venkataramani S., Truntzer J., Coleman D. (2013). Thermal stability of high concentration lysozyme across varying pH: A Fourier Transform Infrared study. J. Pharm. Bioallied Sci..

[49] Basheeruddin M., Khan S., Ahmed N., Jamal S. (2022). Effect of pH on Diclofenac–Lysozyme Interaction: Structural and Functional Aspect. Front. Mol. Biosci..

[50] Kudou M., Shiraki K., Fujiwara S., Imanaka T., Takagi M. (2003). Prevention of thermal inactivation and aggregation of lysozyme by polyamines. Eur. J. Biochem..

[51] Nigen M., Croguennec T., Bouhallab S. (2009). Formation and stability of a-lactalbumin-lysozyme spherical particles: Involvement of electrostatic forces. Food Hydrocoll. Oxf..

[52] Matsuda T., Watanabe K., Sato Y. (1982). Interaction Between Ovomucoid and Lysozyme. J. Food Sci..

[53] Kato Y. (1995). Modification of Ovalbumin with Glucose 6-Phosphate by Amino-Carbonyl Reaction. Improvement of Protein Heat Stability and Emulsifying Activity. J. Agric. Food Chem..

[54] Raikos V., Campbell L., Euston S.R. (2007). Rheology and texture of hen’s egg protein heat-set gels as affected by pH and the addition of sugar and/or salt. Food Hydrocoll..

[55] Sydykov B., Oldenhof H., Sieme H., Wolkers W.F. (2017). Hydrogen Bonding Interactions and Enthalpy Relaxation in Sugar/Protein Glasses. J. Pharm. Sci..

[56] Timasheff S.N. (2002). Protein-solvent preferential interactions, protein hydration, and the modulation of biochemical reactions by solvent components. Proc. Natl. Acad. Sci. USA.

[57] Satoshi A., Hiroki I., Shin-Ichi T., Mitsuhiro H. (2018). Sugar-Mediated Stabilization of Protein Against Chemical or Thermal Denaturation. J. Phys. Chem. B.

[58] Kulmyrzaev A., Cory Bryant A., Mcclements D.J. (2000). Influence of Sucrose on the Thermal Denaturation, Gelation, and Emulsion Stabilization of Whey Proteins. J. Agric. Food Chem..

[59] Beg I., Minton A.P., Islam A., Hassan M.I., Ahmad F. (2016). The pH Dependence of Saccharides’ Influence on Thermal Denaturation of Two Model Proteins Supports an Excluded Volume Model for Stabilization Generalized to Allow for Intramolecular Electrostatic Interactions. J. Biol. Chem..

[60] Arntfield S., Ismond M., Murray E. (1990). Thermal analysis of food proteins in relation to processing effects. Therm. Anal. Foods.

[61] Sow L.C., Yang H. (2015). Effects of salt and sugar addition on the physicochemical properties and nanostructure of fish gelatin. Food Hydrocoll..

[62] Iwashita K., Inoue N., Handa A., Shiraki K. (2015). Thermal aggregation of hen egg white proteins in the presence of salts. Protein J..

[63] Zhu W.K., Luo X.G., Lin X.Y., He J. (2011). Effects of Ca2+ Concentration on Calcium Carbonate Crystallization in Egg White Protein Solution. Adv. Mater. Res..

[64] Guo F., Friedman J.M. (2009). Charge Density-Dependent Modifications of Hydration Shell Waters by Hofmeister Ions. J. Am. Chem. Soc..

[65] Vardhanabhuti B., Foegeding E.A. (2008). Effects of dextran sulfate, NaCl, and initial protein concentration on thermal stability of β-lactoglobulin and α-lactalbumin at neutral pH. Food Hydrocoll..

[66] Stavropoulos P., Thanassoulas A., Nounesis G. (2018). The effect of cations on reversibility and thermodynamic stability during thermal denaturation of lysozyme. J. Chem. Thermodyn..

[67] Salis A., Ninham B.W. (2014). Models and mechanisms of Hofmeister effects in electrolyte solutions, and colloid and protein systems revisited. Chem. Soc. Rev..

[68] Schwierz N., Horinek D., Netz R.R. (2010). Reversed anionic Hofmeister series: The interplay of surface charge and surface polarity. Langmuir Acs J. Surf. Colloids.

[69] Burgess D.J. (1990). Practical analysis of complex coacervate systems. J. Colloid Interface Sci..

[70] Schneider C.P., Shukla D., Trout B.L. (2011). Arginine and the Hofmeister Series: The role of ion-ion interactions in protein aggregation suppression. J. Phys. Chem. B.

[71] Sheng L., Ye S., Han K., Zhu G., Ma M., Cai Z. (2019). Consequences of phosphorylation on the structural and foaming properties of ovalbumin under wet-heating conditions. Food Hydrocoll..

[72] Jin H., Li P., Jin Y., Sheng L. (2021). Effect of sodium tripolyphosphate on the interaction and aggregation behavior of ovalbumin-lysozyme complex. Food Chem..

[73] Tanaka F., Forster L.S., Pal P.K., Rupley J.A. (1975). The circular dichroism of lysozyme. J. Biol. Chem..

[74] Kelly S., Jess T., Price N.C. (2005). How to study proteins by circular dichroism. Biochim. Biophys. Acta.

[75] Jha A.V.P. (2017). Sustained Stability and Activity of Lysozyme in Choline Chloride Against pH Induced Denaturation. ACS Sustain. Chem. Eng..

[76] Relkin P., Launay B., Eynard L. (1993). Effect of Sodium and Calcium Addition on Thermal Denaturation of Apo-α-Lactalbumin: A Differential Scanning Calorimetric Study. J. Dairy Sci..

